# Association between cam-type femoroacetabular impingement and osteitis pubis in non-athletic population on magnetic resonance imaging

**DOI:** 10.1186/s13018-019-1368-6

**Published:** 2019-10-22

**Authors:** Ayşe Serap Akgün, Mehmet Agirman

**Affiliations:** 1 Faculty of Medicine, Department of Physical Medicine and Rehabilitation, Istanbul Medipol University, TEM Avrupa Otoyolu Goztepe Cikisi, No 1, Bagcilar, 34214 Istanbul, Turkey; 2 Faculty of Medicine, Department of Radiology, Istanbul Medipol University, TEM Avrupa Otoyolu Goztepe Cikisi, No 1, Bagcilar, 34214 Istanbul, Turkey

**Keywords:** Femoroacetabular impingement, Osteitis pubis, Magnetic resonance imaging

## Abstract

**Background:**

Osteitis pubis (OP) is a common source of groin and extra-articular hip pain and is associated with intra-articular hip pathology. In this study, we aimed to determine the prevalence of osteitis pubis on magnetic resonance imaging (MRI) in non-athletic patients with cam-type femoroacetabular impingement (FAI).

**Methods:**

This retrospective cross-sectional study included 178 subjects: 90 patients with cam-type FAI diagnosed by MRI and 88 subjects used as a control group. Additionally, their MRI data were analyzed for the characteristics of osteitis pubis, with severity graded from minimal to severe on a four-point scale.

**Results:**

A total of 98 patients and 88 controls were studied. Seventy-two males (80%) and 18 females (20%) were the patient group, whereas 71 males (80.68%) and 17 females (19.32%) were the control group. The mean alpha angle of the patients with FAI was 65.8 ± 3.3° in the right side and 66.2 ± 3.2° in the left side, whereas in the control group, it was 47 ± 5.6° in the right side and 47.8 ± 5.2° in the left side. Alpha angle measurements were significantly higher in the patient group than the control group (*p <* 0.001). A statistically significant increase in the prevalence of osteitis pubis was found in patients with cam-type FAI (45.56%) compared to control subjects (5.68%) (*p* < 0.001).

**Conclusions:**

This study demonstrated that the frequency of osteitis pubis was increased in non-athletic patients with FAI syndrome. Further studies are required to determine whether these findings reflect the clinical symptoms in patients with hip pain.

## Background

Femoroacetabular impingement (FAI) is one of the causes of intra-articular hip pain observed in young adults [1–3]. The cause of pain in FAI is abnormal contact between the femoral head and the acetabulum during joint movement, especially in flexion, resulting in a labrum tear, cartilage damage, and in advanced severe cases [4, 5]. Pain is located in the hip and groin, which is caused by flexion and internal rotation, extending over the trochanters. There is more restriction in internal rotation of the hip joint and is more severe than in other range of motions (ROM). Osteitis pubis (OP) is a common source of groin and extra-articular hip pain and is associated with intra-articular hip pathology [6–9]. Some research has shown that restricted hip ROM results in hip stiffness that induces increased stress over the superior pubic ramus and pubic symphysis, and results in a bony stress response [10, 11]. This association is described mostly in elite-level athletes. Unlike others, this study aimed to search for the coexistence of osteitis pubis and cam-type FAI in a non-athletic population.

## Methods

This retrospective study was performed in symptomatic FAI patients (aged 18–60 years) experiencing hip pain for more than 3 months, and the impingement test results were positive and compatible with the diagnosis. They were referred to the radiology department of our institution to undergo hip MRI of both joints between January 2017 and July 2018. Pertinent demographic and clinical history was obtained from the medical files. All patients signed written informed consent forms to participate in the study, which was approved by the local ethics committee. This study was conducted in accordance with the principles of the Declaration of Helsinki.

Exclusion criteria: (1) The diagnosis was not clear or presence of unilateral hip pain, (2) severe osteoarthritis, (3) inflammatory diseases (rheumatoid arthritis, ankylosing spondylitis, etc.), (4) hip dysplasia, (5) history of hip joint and pelvic injury and previous hip surgery, (6) history of having regularly sportive activity, (7) MRI images are not clear and sequences are not complete, and (8) over coverage of the acetabulum. In total, the consecutive MRI reports and images from 247 patients were collected. One hundred fifty-seven cases were excluded because their diagnosis was not FAI (*n* = 109) and had previous hip surgery (*n* = 31), rheumatoid arthritis (*n* = 6), hip dysplasia (*n* = 4), elite athletes (*n* = 2), pincer type FAI (*n* = 3), and mixed type FAI (*n* = 2). From the remaining sample set, 90 patients’ MRI scans were reviewed to confirm the presence of FAI. The age- and sex-matched control group included subjects who had no known diseases affecting the proximal femur and no symptoms of FAI and underwent pelvic MRI due to several purposes (e.g., pelvic diseases, trauma); the hip was included in the scan range. The exclusion criteria included malformation, fracture, tumor in the proximal femur, over coverage of the acetabulum, and a bony bump at the femoral head-neck junction.

For this purpose, the picture archiving and communication system (PACS, General Electric, Chicago, IL, USA) was used. All MRI examinations were performed using Ingenia 1.5 Tesla MRI scanners (Philips Best, Netherlands). The patients were placed in the supine position while their hip joints were maintained in a neutral position using a phased array coil. Sequences were obtained in 4-mm slices, T1-weighted coronal and axial images from the body of the pubis to the coxae (TR, 621 ms; TE, 7 ms), T2-weighted coronal from sacrum to pubis (TR, 3500 ms; TE, 80 ms), and T2-weighted with FS axial to the body of the pubis (TR, 3500 ms; TE, 80 ms). All the scans were reviewed independently for FAI and radiographic evidence of OP by two observers (first was the radiologist and experienced with musculoskeletal MRI for 8 years). When their individual findings did not consort with each other, they reached a consensus through discussion. Firstly, all images were evaluated in terms of an osseous bump, labral tears, cartilage lesions, muscle impairment, paralabral cyst, and herniation pits. Previous studies have shown that there is a significant association between the increased alpha angle and the presence of cam-type FAI. An alpha angle greater than 55° is indicative of a cam-type FAI diagnosis [12, 13]. Alpha angles were measured by the radiologist twice for each patient using the method described by Nötzli et al.’s [14] criteria in T1A axial sequences, and the average values were used for statistical purposes. The alpha angle of the hip was defined as the angle between two intersecting lines at the center of the femoral head. Using a best-fit circle, which was drawn outlining the femoral head, the first line was extended from the center of the femoral head to the midpoint of the femoral neck. The second line was drawn from the center of the femoral head to the deviation of the femoral neck from the circle drawn around the femoral head. All patients were then evaluated for osteitis pubis and graded by two observers. Typical MRI signs in OP are bone marrow edema, osseous irregularity, bone resorption, periosteal reaction, osteophytes, fatty degeneration, subchondral cyst, tendon lesions, and fluid in the symphysis [15, 16]. Assessment of osteitis pubis on a four-point scale was evaluated as follows: 0 (normal), 1 (minimal-mild), 2 (moderate), and 3 (severe). The age- and sex-matched control group was also assessed for alpha angle and radiographic findings of osteitis pubis.

All qualitative variables were reported as frequencies and percentages. The continuous variables were expressed as mean ± standard deviation. Nonparametric tests were performed because the data in the control and patient groups were not normally distributed. The Mann-Whitney *U* test was used for the statistical difference between the groups. SPSS® version 21 (IBM Corp., Armonk, USA) was used for the statistical analysis. A *p* value of < 0.05 was considered to be statistically significant.

## Results

**Fig. 1:**
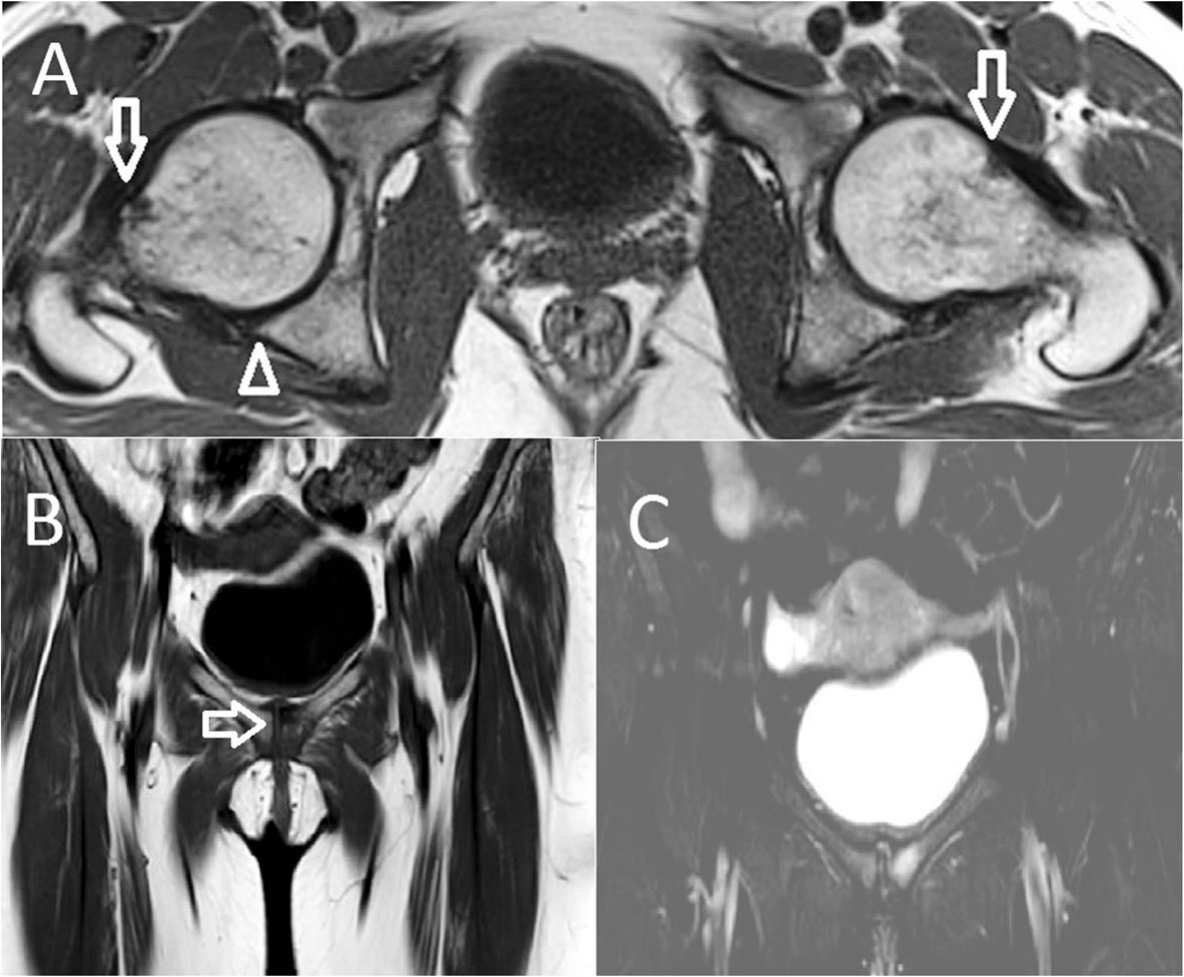
Fifty-year-old female who had a 3-month history of pubic pain with diagnosed femoroacetabular impingement. **a** Axial T1-weighted MR image shows bilateral osseous bumps at the femoral head and neck junctions (thin arrows) and bilateral synovial herniation pits on the left and right side (arrow) and minimal osteophytes (arrowheads). **b** Coronal T1-weighted MR image with osseous irregularity on both sides of the symphysis pubis (arrow). **c** Coronal fat-saturated T2-weighted MR image shows mild osteitis pubis with left-side predominant subchondral bone marrow edema

**Fig. 2:**
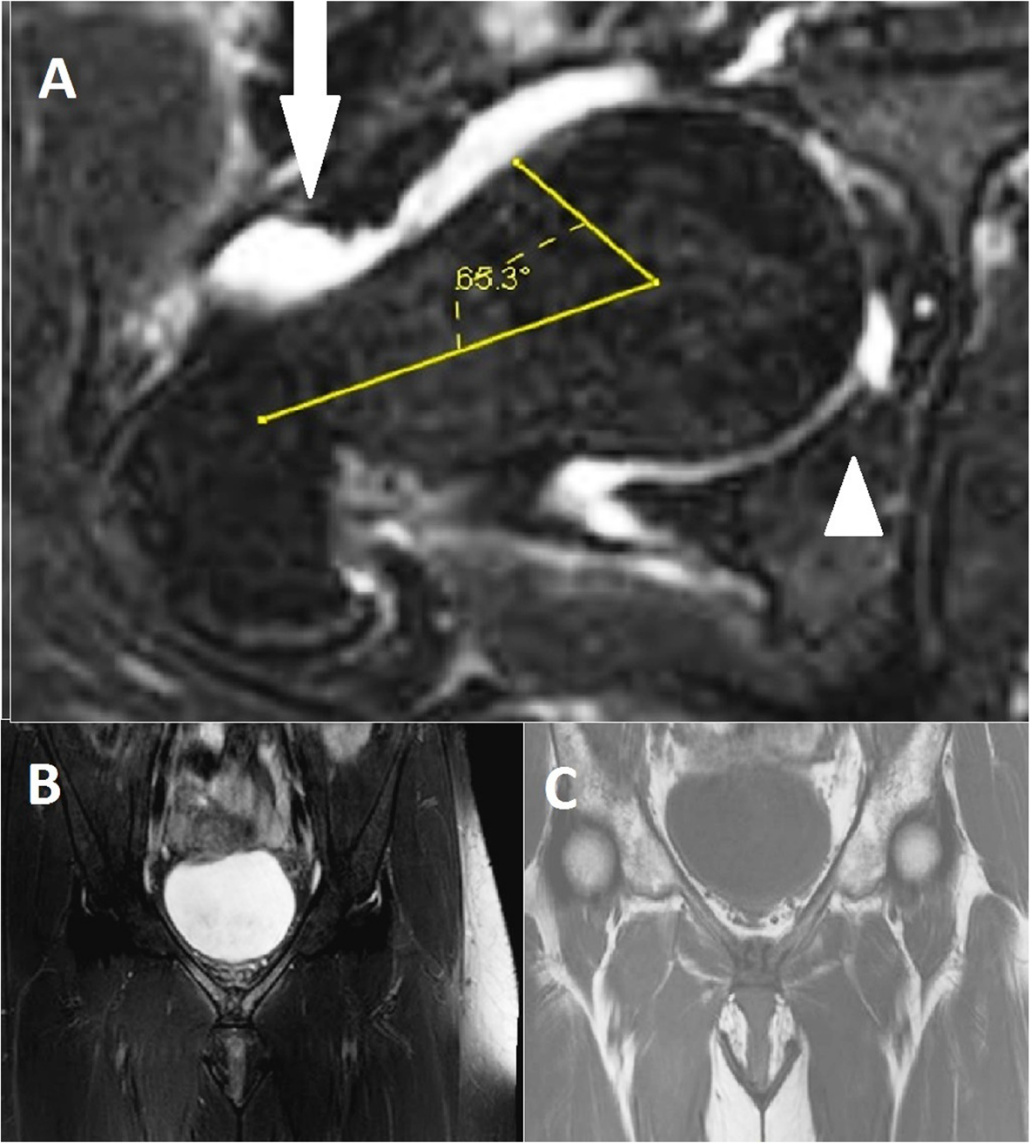
Forty-five-year-old female with chronic hip pain and diagnosed femoroacetabular impingement. **a** Axial fat-saturated T2-weighted MR image shows an increased alpha angle (65.3°), paralabral cyst (solid arrow), and labral tear (arrow head). **b** Coronal T1-weighted MR image showing acute-on-chronic osteitis pubis with osseous irregularity, degenerative changes, and hypointensity on both sides of the symphysis pubis. **c** Coronal fat-saturated T2-weighted image of MR showing diffuse bone marrow and soft tissue edema

**Fig. 3:**
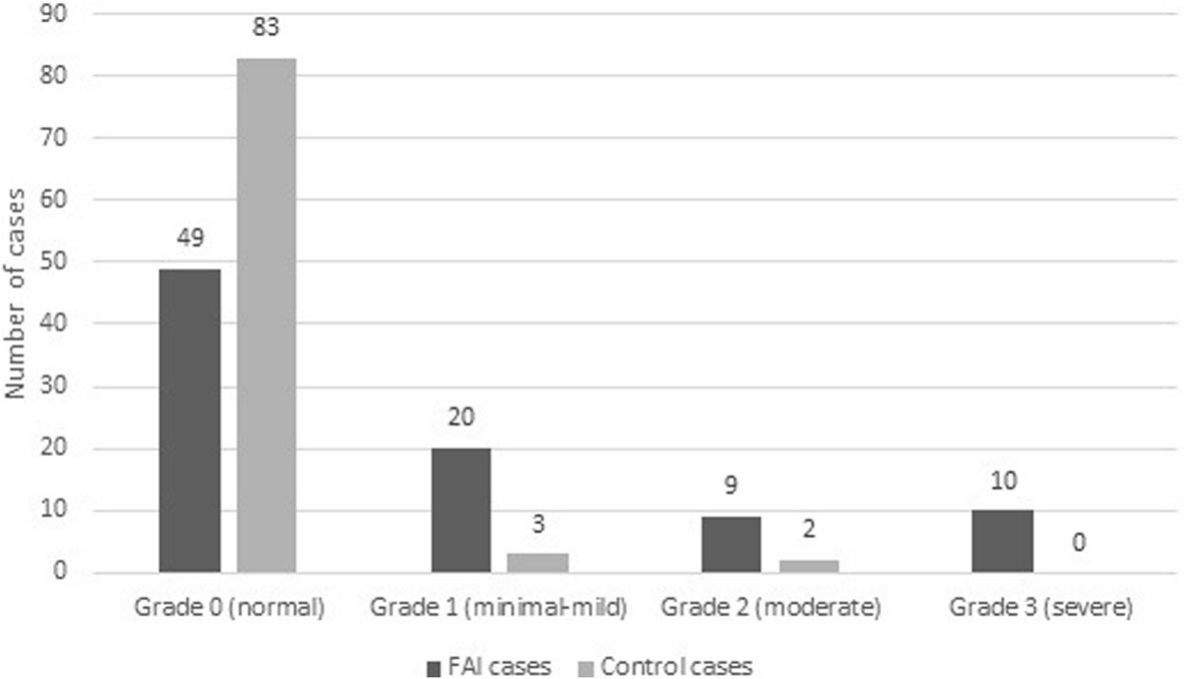
Severity of osteitis pubis based on a four-point scale from grades 0–3, Grade 0 = normal, grade 1 = minimal-mild, grade 2 = moderate, and grade 3 = severe

Ninety patients with cam-type FAI diagnosed by MRI and 88 age- and sex-matched control subjects were evaluated in this study. Seventy-two males (80%) and 18 females (20%) made up the patient group, whereas 71 males (80.68%) and 17 females (19.32%) made up the control group. The mean age of the patient group was 38.58 ± 9.78, and the mean age of the control group was 39.8 ± 9.8. There were no statistically significant differences in mean ages or genders between the patient and control groups (*p* > 0.05) (Table 1). MRI findings in the 90 patients included labral tears (80%), cartilage lesions (60%), paralabral cysts (2.2%), muscle impairment (13.3%), and synovial herniation pits (11%), and in the control group, only labral tears (5%) and synovial herniation pits (4.5%) were seen. The mean alpha angle of the patients with FAI was 65.8 ± 3.3° in the right side and 66.2 ± 3.2° in the left side, whereas in the healthy group, it was 47 ± 5.6° in the right side and 47.8 ± 5.2° in the left side. There was no significant difference between the right and the left sides in each group. Alpha angle measurements were significantly higher in the patient group than the control group (*p* < 0.001). A statistically significant increase in the prevalence of osteitis pubis was found in patients with FAI compared with the age-matched control group, with a prevalence of 45.56% in the FAI group compared to 5.68% in the control group (*p* < 0.001) (Table 2). The odds ratio (OR) for the presence of OP in FAI cases was 13.89 (95% confidence interval, 5.14–37.50), meaning that the probability of occurrence of OP in cam-type FAI was 13.89 times more than the control group. In the patient group, grade 1 osteitis pubis was seen in 20 patients (22.2%), grade 2 osteitis pubis in 9 patients (10%), and grade 3 osteitis pubis in 10 patients (11.1%). In the control group, grade 1 osteitis pubis was seen in 3 subjects (3.4%) and grade 2 osteitis pubis in 2 subjects (2.27%). No control cases were diagnosed with grade 3 osteitis pubis. Figures 1 and 2 show MRI views of two cases with both cam-type femoroacetabular impingement and osteitis pubis. The most common findings in patients with OP in both the FAI and the control groups were osseous irregularity (80%), sclerosis (45%), bone marrow edema (24%), osteophyte (18%), and fatty degeneration (10%). Figure 3 shows the distribution of severity of osteitis pubis.

**Table 1:** Demographic values of the subjects

		FAI patients		Controls		*p*
		*n*	%	*n*	%	
Sex	Male	72	(80.00)	71	(80.68)	0.909
	Female	18	(20.00)	17	(19.32)	
Age (mean ± s.d. and median showed)		38.58 ± 9.78	37.00	39.89 ± 9.87	38.00	0.086

**Table 2:** Presence of osteoitis pubis in two groups

		FAI patients (*n*:90)		Controls (*n*:88)		*p*
		*n*	%	*n*	%	
Osteoitis pubis	Absent	49	(54.44)	83	(94.32)	< 0.001
	Present	41	(45.56)	5	(5.68)

## Discussion

In patients with hip and groin pain, the medical history, the location, and the physical examination findings are important to distinguish between intra- and extra-articular sources. OP is a cause of extra-articular groin pain, whereas FAI, which causes labral tears, is a cause of intra-articular pain. OP is a noninfectious inflammation of the pubic symphysis and surrounding muscle insertions. Activities such as kicking, turning, twisting, and pivoting cause overuse syndromes, leading to cumulative microtrauma and altering the biomechanics of the symphysis pubis and surrounding soft tissues. This results in symphyseal instability and OP lesions [17, 18]. On MRI, bilateral or less often asymmetric subchondral plate marrow edema extending from anterior to posterior is an acute finding, although a periosteal reaction, bone resorption, irregular contour of articular surfaces, osteophytes, and subchondral cyst formation are chronic phase findings. Cam-type FAI is characterized by the presence of a spherical portion of the femoral head-neck junction. In cam-type FAI, flexion, adduction, and internal rotation lead to labral compression and labral tear, resulting in pain in the hips [19]. The first joint motion to be affected is internal rotation and adduction. To reduce the pain, especially with the reduction of internal rotation, stress over the symphysis and surrounding soft tissues is increased, resulting in OP [10, 11]. Howse [20] first described this mechanistic link in 1964. The association between FAI and OP has also been shown in many studies, especially with elite athletes [6, 8–10]. Larson et al. [7] found a 54% prevalence of radiographic OP in high-level collegiate football players with FAI on hip radiographs. A cadaveric study [21] showed that the cam morphology is associated with decreased internal rotation and a significant increase in rotational motion at the pubic symphysis. Unlike in previous studies, we evaluated patients with cam-type FAI who had been diagnosed with routine MRI sequences with hip pain in a nonathletic normal population. However, it is not known whether there is an additional groin or pubic pain in the cases with symptomatic FAI that showed a higher prevalence of OP than the control groups. The current findings support previous studies’ results especially regarding elite athletes.

Our study has several limitations. First, the cases of this research were selected from just one hospital, the sample range was narrow, the sample size was not big enough, and there may be some bias. Second, this was a retrospective study; we could not obtain all the clinical information, physical examination findings, activity frequency, and pain localization. Third, we used the alpha angle as a radiological measurement to assess the final diagnosis of cam-type FAI. Although the normal value of the alpha angle is still controversial, we have accepted it pathologically to be above 55°, as stated by Pfirrmann et al. [13]. Another limitation was that during the assessment of osteitis pubis, the observers were not blind to the patients who had femoroacetabular impingement, leading to bias. Last, osteitis pubis’ diagnosis was made on the MRI findings alone and graded in a four-point scale, without knowledge of a history of pubic tenderness or pubis pain, childbirth, previous injuries, and clinical examination. In the literature, the absence of a standardized test for osteitis pubis grading is a major drawback. Future research is necessary to identify accurate and reproducible radiologic findings to diagnose and grade the osteitis pubis not only in elite athletes but also in the normal population.

## Conclusions

The frequency of osteitis pubis was increased in patients diagnosed with cam-type femoroacetabular impingement in non-athletic population. These findings support the need to identify the pathophysiology of this co-existence. This association with larger studies involving larger masses should be assessed with clinical and radiological findings and should be identified and examined with the prognosis of OP in both normal populations and athletes. Clinicians must be aware of this association when diagnosing and treating patients with femoroacetabular impingement in the general population as in elite athletes.

## Data Availability

The data supporting the conclusions of this article are included within the article.

## Abbreviations

FAI: Femoroacetabular impingement
MRI: Magnetic resonance imaging
OP: Osteitis pubis
ROM: Range of motion

## References

[1] Parvizi J, Campfield A, Clohisy JC, Rothman RH, Mont MA (2006). Management of arthritis of the hip in the young adult. J Bone Joint Surg (Br).

[2] Ganz R, Parvizi J, Beck M, Leunig M, Nötzli H, Siebenrock KA (2003). Femoroacetabular impingement: a cause for osteoarthritis of the hip. Clin Orthop Relat Res.

[3] Clohisy JC, Nunley RM, Otto RJ, Schoenecker PL (2007). The frog-leg lateral radiograph accurately visualized hip cam impingement abnormalities. Clin Orthop Relat Res.

[4] Ganz R, Gill TJ, Gautier E, Ganz K, Krügel N, Berlemann U (2001). Surgical dislocation of the adult hip a technique with full access to the femoral head and acetabulum without the risk of avascular necrosis. J Bone JointSurg [Br].

[5] Leunig M, Fraitzl CR, Ganz R (2002). Early damage to the acetabular cartilage in slipped capital femoral epiphysis. Therapeutic consequences. Orthopade.

[6] Matsuda DK (2010). Endoscopic pubic symphysectomy for recalcitrant osteitis pubis associated with bilateral femoroacetabular impingement. Orthopedics..

[7] Larson CM, Sikka RS, Sardelli MC, Byrd JW, Kelly BT, Jain RK (2013). Increasing alpha angle is predictive of athletic related “hip” and “groin” pain in collegiate National Football League prospects. Arthroscopy..

[8] Lohan DG, Seeger LL, Motamedi K, Hame S, Sayre J (2009). Cam-type femoral-acetabular impingement: is the alpha angle the best MR arthrography has to offer?. Skelet Radiol.

[9] Larson CM, Pierce BR, Giveans MR (2011). Treatment of athletes with symptomatic intra-articular hip pathology and athletic pubalgia/sports hernia: a case series. Arthroscopy..

[10] Verrall GM, Hamilton IA, Slavotinek JP, Oakeshott RD, Spriggins AJ, Barnes PG (2005). Hip joint range of motion reduction in sports-related chronic groin injury diagnosed as pubic bone stress injury. J Sci Med Sport.

[11] Fricker PA (1997). Reviews management of groin. Br J Sports Med.

[12] Ratzlaff C, Zhang C, Korzan J, Josey L, Wong H, Cibere J (2016). The validity of a non-radiologist reader in identifying cam and pincer femoroacetabular impingement (FAI) using plain radiography. Rheumatol Int.

[13] Pfirrmann CW, Mengiardi B, Dora C, Kalberer F, Zanetti M, Hodler J (2006). Cam and pincer femoroacetabular impingement: characteristic MR arthrographic findings in 50 patients. Radiology..

[14] Nötzli HP, Wyss TF, Stoecklin CH, Schmid MR, Treiber K, Hodler J (2002). The contour of the femoral head-neck junction as a predictor for the risk of anterior impingement. J Bone Joint Surg Br.

[15] Khan W, Zoga AC, Meyers WC (2013). Magnetic resonance imaging of athletic pubalgia and the sports hernia: current understanding and practice. Magn Reson Imaging Clin N Am.

[16] Paajanen H, Heikkinen J, Hermunen H, Airo I (2005). Successful treatment of osteitis pubis by using totally extraperitoneal endoscopic technique. Int J Sports Med.

[17] Murar J, Birmingham PM, Nho S, Leunig M, Larson CM, Bedi A, Kelly B (2014). Osteitis pubis. Hiparthroscopyandhip joint preservation surgery.

[18] Rodriguez C, Miguel A, Lima H, Heinrichs K (2001). Osteitis pubis syndrome in the professional soccer athlete: a case report. J Athl Train.

[19] Ganz R, Leunig M, Leunig-Ganz K, Harris WH (2008). The etiology of osteoarthritis of the hip: an integrated mechanical concept. Clin Orthop Relat Res.

[20] Howse AJ (1964). Osteitis pubis in an olympic road walker. Proc R SocMed.

[21] Birmingham PM, Kelly BT, Jacobs R, McGrady L, Wang M (2012). The effect of dynamic femoro acetabular impingement on pubic symphysis motion: a cadaveric study. Am J Sports Med.

